# Supplementary material for: Imprecision nutrition? Duplicate meals result in unreliable individual glycemic responses measured by continuous glucose monitors across four dietary patterns in adults without diabetes
Source: medRxiv. 2023 Dec 11:2023.06.14.23291406. Originally published 2023 Jun 15. Preprint. [Version 2] doi: 10.1101/2023.06.14.23291406 (PMC10371100; doi:10.1101/2023.06.14.23291406)

medRxiv preprint doi: https://doi.org/10.1101/2023.06.14.23291406; this version posted December 11, 2023. The copyright holder for this preprint (which was not certified by peer review) is the author/funder, who has granted medRxiv a license to display the preprint in perpetuity. This article is a US Government work. It is not subject to copyright under 17 USC 105 and is also made available for use under a CC0 license.

Online Supplemental File

Imprecision nutrition? Duplicate meals result in unreliable individual glycemic responses measured by continuous glucose monitors across four dietary patterns in adults without diabetes

*Aaron Hengist[1], Jude Ong[1], Katherine McNeel[1], Juen Guo[1], Kevin D Hall[1]**

Supplemental Figures

Supplemental Figure 1. Mean ±SD difference and individual comparisons of duplicate meals organized by participant using A) Abbott and B) Dexcom devices. Each data point is the within-participant iAUC difference between duplicate meals. Mean ±SD and individual comparisons of duplicate meals ordered by meal pairing (across all participants) using C) Abbott and D) Dexcom CGMs. Each data point is a duplicate meal eaten in week 2 minus the same meal eaten in week 1 with data from all participants who consumed that meal (abbott has 42 total meals for comparison across the 14 days of rotating menu, 14 days x 3 meals; dexcom has 63 total meals for comparison across 21 days of rotating menu, 21 days x 3 meals).

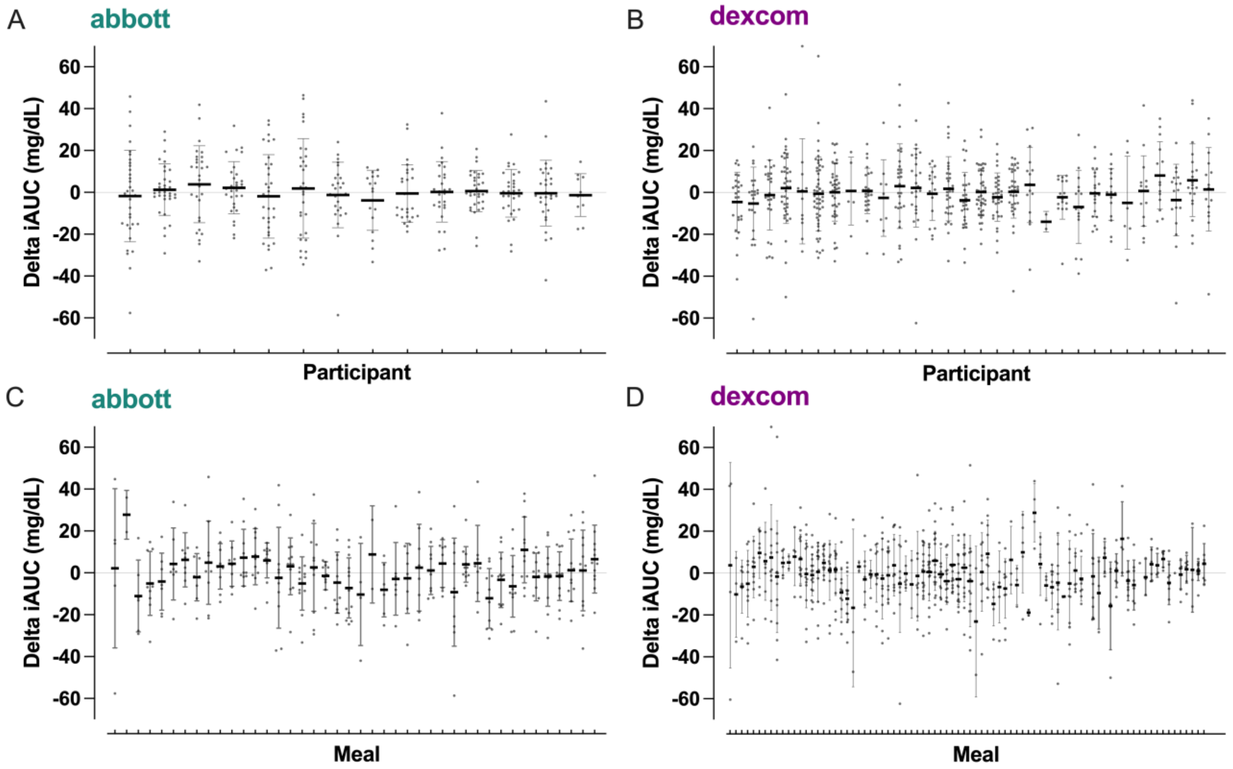

medRxiv preprint doi: https://doi.org/10.1101/2023.06.14.23291406; this version posted December 11, 2023. The copyright holder for this preprint (which was not certified by peer review) is the author/funder, who has granted medRxiv a license to display the preprint in perpetuity. This article is a US Government work. It is not subject to copyright under 17 USC 105 and is also made available for use under a CC0 license.

Supplemental Figure 2. Mean and individual meal responses from lower tertile and upper tertile meals during week 1 and the corresponding comparisons in week 2. Lower tertile meals were significantly higher in week 2 for Abbott (**A**) and Dexcom (**C**) and upper tertile meals were significantly lower in week 2 for Abbott (**B**) and Dexcom (**D**). Dashed lines are mean of all presented meal responses across 2 weeks. iAUC = incremental area under the curve.

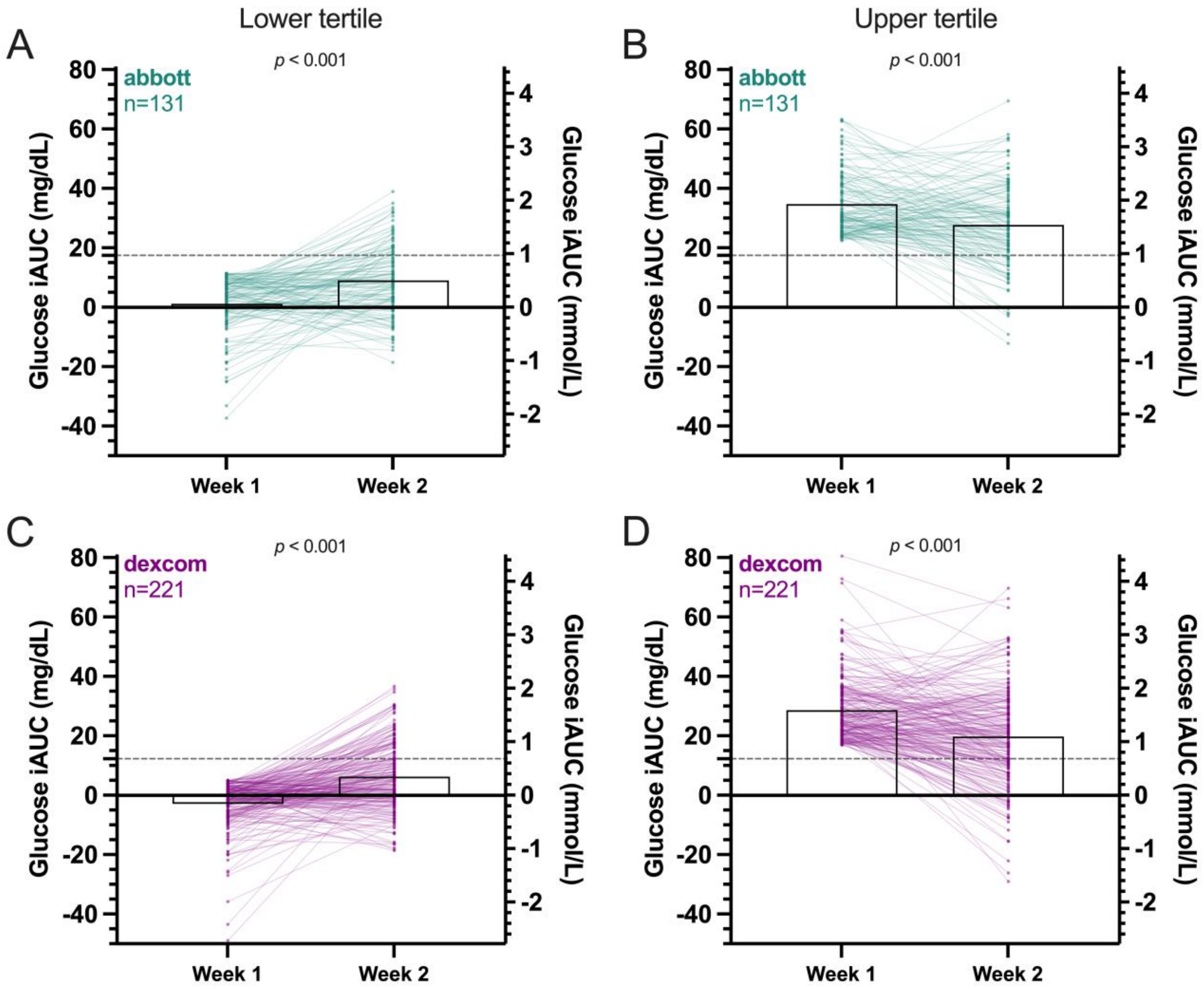

Supplement: 1 [file NIHPP2023.06.14.23291406v2-supplement-1.pdf]
